# Autoimmunity against cytokines: Double strike in autoimmune disease, a historical perspective

**DOI:** 10.7705/biomedica.7570

**Published:** 2024-12-23

**Authors:** Iván Insignares, Luis E. Rodríguez, Óscar Correa-Jiménez, Alberto Alfaro-Murillo, Laura Rincón-Arenas, Andrés Sánchez, Marlon Múnera

**Affiliations:** 1 Facultad de Ciencias de la Salud, Corporación Universitaria Rafael Núñez, Cartagena, Colombia Corporación Universitaria Rafael Núñez Facultad de Ciencias de la Salud Corporación Universitaria Rafael Núñez Cartagena Colombia; 2 Centros Médicos Colsanitas, Bogotá, D. C., Colombia Centros Médicos Colsanitas Centros Médicos Colsanitas Bogotá, D. C. Colombia; 3 Grupo de Investigación en Neumología e Inmunología en Pediatría, Facultad de Medicina, Universidad Nacional de Colombia, Bogotá, D. C., Colombia Universidad Nacional de Colombia Grupo de Investigación en Neumología e Inmunología en Pediatría Facultad de Medicina Universidad Nacional de Colombia Bogotá, D. C. Colombia; 4 División de Inmunología, Departamento de Medicina Interna, Hospital San Juan de Dios, Caja Costarricense de Seguro Social, San José, Costa Rica Hospital San Juan de Dios División de Inmunología, Departamento de Medicina Interna Hospital San Juan de Dios Caja Costarricense de Seguro Social San José Costa Rica; 5 Unidad de Alergias e Inmunología, Cayre IPS, Bogotá, D. C. Colombia Cayre IPS Unidad de Alergias e Inmunología Cayre IPS Bogotá, D. C. Colombia; 6 Grupo de Investigación Nuñista de Medicina-GINUMED, Facultad de Ciencias de la Salud, Corporación Universitaria Rafael Núñez, Cartagena, Colombia Corporación Universitaria Rafael Núñez Grupo de Investigación Nuñista de Medicina-GINUMED Facultad de Ciencias de la Salud Corporación Universitaria Rafael Núñez Cartagena Colombia

**Keywords:** Autoimmunity, cytokines, autoantigens, autoantibodies., autoinmunidad, citocinas, autoantígenos, autoanticuerpos.

## Abstract

Autoimmune responses are characterized by the development of antibodies and the activation of T lymphocytes against self-antigens. This leads to an effector immune response against tissues expressing antigens, which are later recognized by the host immune system.

Host antigens attacked by antibodies are called “autoantigens” and are of different kinds, including receptors, enzymes, and channel proteins. The autoimmune response is potentiated by cytokines that mediate the activation of Th1, Th2, or Th17 lymphocytes. The released cytokines can also be recognized as autoantigens, meaning they can be targets of the autoimmune response. The effects of autoimmunity on cytokines or their receptors are diverse, and the mechanisms of this type of autoimmune response are discussed in this review.

## Resumen

Las reacciones autoinmunitarias se caracterizan por el desarrollo de anticuerpos y la activación de linfocitos T contra autoantígenos. Esto desata una respuesta inmunitaria efectora contra los tejidos que expresan antígenos reconocidos por la autoinmunidad. Los antígenos atacados por anticuerpos se denominan “autoantígenos” y son de diferentes tipos, como receptores, enzimas y proteínas de canal.

La reacción autoinmunitaria es potenciada por citocinas que median la activación de los linfocitos Th1, Th2 o Th17. Las citocinas liberadas también pueden ser reconocidas como autoantígenos y, por lo tanto, pueden ser objetivos de la reacción autoinmunitaria. Los efectos de la autoinmunidad en las citocinas o sus receptores son diversos, razón por la cual los mecanismos de este tipo de reacción autoinmunitaria se discuten en esta revisión.

Cytokines are soluble molecules that contribute to cell communication and induce signals through their respective receptors, which are released by different types of cells of innate and adaptive immunity ^1^. These proteins modulate inflammatory responses and tolerance mechanisms. They can act in an autocrine or paracrine manner, making their effects local or systemic depending on the immune system requirements. In addition, they can induce antagonistic responses such as IL-4 and IL-10, the former favoring the production of IgE antibodies, and the latter inhibiting their production. On the other hand, they can act cooperatively to promote responses that could not be achieved individually, a process known as synergism ^1^^,^^2^.

Cytokines also play an important role in autoimmune responses, where the immune system loses tolerance towards itself, generating effector responses. In this type of disease, antibodies are produced against self-antigens. This type of antibody is usually known as autoantibodies or autoreactive antibodies ^3^. However, the role of autoantibodies against cytokines, a phenomenon with important clinical implications, has only been studied for less than a decade.

Autoimmunity against cytokines is considered a type of immunodeficiency due to errors in the activation of the immune system, secondary to the neutralization of cytokines ^4^^,^^5^. It has been found that this type of autoimmunity affects several cytokines: autoantibodies against cytokines, such as interferons IL-6, IL-2, IL-10, and IL-17 ^6^^-^^8^, and autoantibodies against cytokine receptors, such as the IL-1 alpha subunit receptor, have been reported to date ^8^.

The clinical impact of these autoimmune responses is variable. Autoantibodies against interferons are known to increase susceptibility to bacterial and viral infections. This type of autoimmune response is mainly described in diseases such as systemic lupus erythematosus ^9^, although it has also been reported in COVID-19. In the latter, the autoantibodies are considered to precede the development of the autoimmune response ^10^.

**Figure 1.**
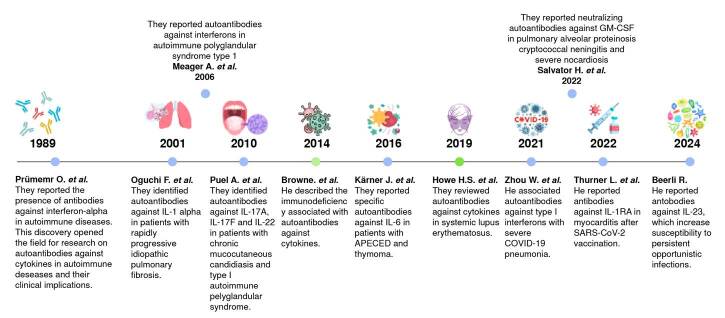
Historical progress about autoimmune response against cytokines APECED: autoimmunepolyendocrinopathy-candidiasis-ectodermal dystrophy

Figure 1 shows the timeline with milestones of anti-cytokine autoimmunity.

## History

The discovery of cytokines and the study of autoimmunity date back to the late 19^th^ and early 20^th^ centuries, with the concept of “antitoxins” proposed by Emil von Behring and Shibasaburo, who discovered that the administration of serum from immune patients to non-immunized patients infected with tetanus produced an adequate response against the infection ^11^. This concept is now known as passive immunity. It earned Emil von Behring the first Nobel Prize in Medicine and made him a pioneer in the study of immunology ^11^.

Later, in 1957, Isaacs and Lindemann described “interferon” while studying the immune response to viruses ^12^. In 1967, Robinson’s group discovered colony-stimulating factors ^13^, and afterward, in 1969, Dumonde’s group coined the term “lymphokine” for cell mediators secreted by lymphocytes, leading to “monokine” for those produced by monocytes ^14^. In 1974, Cohen’s research group coined “cytokines” to refer to cellular mediators produced by various cells ^15^. Then, in 1989, antibodies to interferon alpha (IFN-a) were first reported, paving the way for the study of autoantibodies ^16^.

Figure 1 shows the timeline of major subsequent discoveries, including autoantibodies to type I interferon in patients with severe COVID-19 pneumonia and new disease associations published this year. 

## Anti-cytokine autoantibodies: The fundamental basis of phenocopies of inborn errors of immunity

Anti-cytokine autoantibodies have important clinical implications in several: they have relevant descriptions in the pathophysiology of diverse disorders in rheumatology ^1^, pulmonology ^17^, and infectious diseases, among others ^18^.

However, one of the areas of exponential discovery in recent years has been the field of inborn errors of immunity, formerly known as primary immunodeficiencies ^5^. Various studies published in the context of severe COVID-19 have shown the significant role of autoantibodies against type 1 interferon, requiring intensive care unit treatment, or leading to death, which is more frequent in male and young patients ^6^^-^^19^. This type of condition is referred to as phenocopy of inborn errors of immunity because, despite the lack of germline genetic variants causing specific phenotypes of primary immunodeficiencies, additional arrangements, such as somatic mutations or autoantibodies, induce defects in the immune mechanisms involved in these pathways, generating the same phenotype ^20^.

Despite their recent prominence in the context of COVID-19, anticytokine autoantibodies have classically been described as important pathophysiological factors in inborn errors of immunity. In fact, since its 2015 edition ^6^, the phenotypic classification of inborn errors of immunity of the International Union of Immunological Societies (IUIS) includes a subgroup of defects associated with autoantibodies in the category of phenocopies. The new members of this group are the anti-interferon type 1 autoantibodies in the context of severe COVID-19 ^19^^,^^21^. Autoantibodies against other cytokines have classically been reported in this category ^21^.

Anti-interferon gamma autoantibodies have been described in the pathophysiological mechanisms of adult-onset immunodeficiency, with susceptibility to mycobacterial infections and increased susceptibility to fungal infections, *Salmonella* spp. or varicella zoster ^22^. Similarly, autoantibodies against the Th 17 profile cytokines IL-17, IL-22, and IL-23 have been associated with chronic mucocutaneous candidiasis ^22^, a condition usually related with different variants, mechanisms, and inheritance patterns in the following genes: *AIRE, IL17F, IL17RA, IL17RC, STAT1, STAT3, DOCK8, TYK2, ZNF341, PGM3, CARD11, RORC, ACT1, MAPK8, IL12, IL12B, IL12RB1, CARD9*, and *CLEC7A*^23^.

Anti-IL6 is within the same classification as anti-cytokine autoantibodies that induce a greater susceptibility to infections. Patients with IL-6 autoantibodies tend to develop pyogenic infections due to staphylococci and pneumococci ^16^^,^^24^.

As previously described, inborn errors of immunity go beyond increased susceptibility to infection and encompass several features grouped under the category of immune dysregulation ^19^. Such manifestations have also been associated with phenocopies. Anti-interferon alpha autoantibodies have been described in several autoimmune diseases, including systemic lupus erythematosus, type 1 diabetes, and thyroid disease ^23^^,^^25^. Another non-infectious phenotype associated with anti-cytokine autoantibodies is pulmonary alveolar proteinosis, a rare respiratory syndrome characterized by the accumulation of surfactant lipoproteins in the alveoli, with implications for the lung health of affected patients ^25^.

This condition occurs primarily in patients with mutations in the GM-CSF (Granulocyte-Macrophage Colony-Stimulating Factor) receptor, who develop the most severe form of the disease, causing respiratory failure and death in the first days of life ^26^. In patients with anti-GM-CSF autoimmunity, these neutralizing autoantibodies cause alveolar macrophage dysfunction and reduced surfactant clearance, resulting in pulmonary alveolar proteinosis ^27^. Pulmonary alveolar proteinosis has also been described in patients with GATA2-deficiency ^28^, immunomodulatory therapy, or hematological malignancies ^22^.

The mechanisms underlying the development of anti-cytokine autoantibodies as phenocopies of inborn errors of immunity are not fully understood. Bodansky *et al*. ^29^ have recently documented that in the context of patients with severe COVID-19, anti-IFN-a autoantibodies may be due to an inborn error of immunity, specifically related to an NFKB2 haploinsufficiency. Increased availability of anti-cytokine autoantibody measurements and a greater awareness of their clinical implications will probably lead to a better understanding of the intricate mechanisms involved in autoimmunity development and how they can be clinically exploited to benefit the patients.

## Autoimmunity against interferons

Interferons are a subgroup of cytokines with a marked ability to interfere with viral infections but also have other immunomodulatory functions ^30^. Three families of interferons are known:

- *Type I interferons:* Include IFN-a, IFN-(3, IFN-co, IFN-e, IFN-k, IFN-õ, and IFN-t. All are mainly produced by dendritic and plasmacytoid cells and act in innate and adaptive immunity.
- *Type II interferons:* Also known as IFN-y. They are produced by natural killer (NK) cells of the innate immune system and by T cells in the adaptive immune system. They play an important role in the response to intracellular pathogens, the differentiation and activation of macrophages, and the production of cytokines. Phagocytes induce the secretion of IL-12, which binds to NK or T cell receptors and triggers the secretion of IFN-y, which is itself an IL-12 activator, creating a positive feedback loop.
- *Type III interferon:* Also known as IFN-A. It is produced by almost all cells but acts only on epithelial surfaces due to the restriction of its receptors ^31^.

These three families of interferons act through the JAK/STAT pathway, the first is the sequentially active Janus kinase family (JAK and TYK), and the second is the signal transducer and activator of transcription (STAT).

To illustrate the general pattern of the interferon-mediated JAK/STAT pathway, we will use monocytes as a cellular example because they have a significant expression of IFN receptors. In this case, type I interferons bind to the IFN-a receptor (IFNaR), which consists of two subunits, IFNaRI and IFNaR2. When interferon binds, it induces the phosphorylation of TYK2 and JAK1, which sequentially leads to the phosphorylation of STAT1/STAT2. The phosphorylated STAT1/STAT2 complex homodimerizes and translocates to the nucleus to initiate transcription of interferon-dependent genes. This process leads to macrophage differentiation and increased secretion of pro- inflammatory cytokines such as TNF-a and IL-12 ^32^^,^^33^.

**Figure 2.**
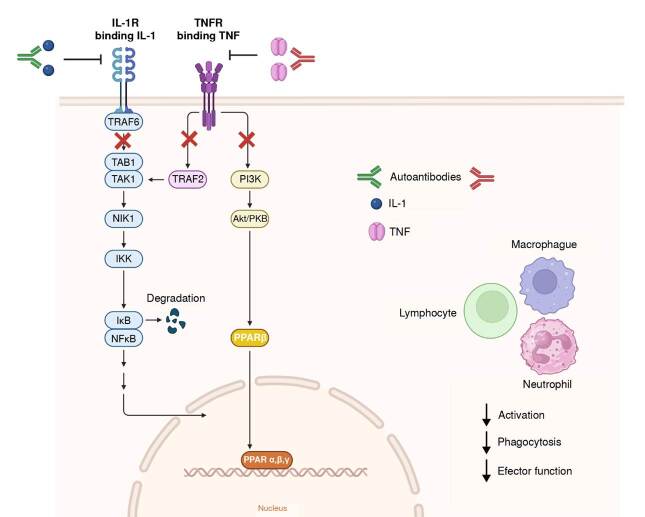
Autoimmunity against cytokines and their receptors plays a pivotal role by reducing biological activity in several cell populations, such as macrophages, neutrophils, and lymphocytes. This autoimmune response also down-regulates immune activity against viral infections.

*In vitro* studies have shown that anti-IFN-y autoantibodies can block downstream mediators of IFN-y, such as phosphorylated STAT1, TNF-a, and IL-12 production. The gene expression response to IFN-y clearly shows that these autoantibodies interfere with the natural inflammatory response to mycobacterial and viral infection (figure 2) ^33^.

Antibodies against interferons block the response induced by these cytokines by interfering with the binding between interferon and its receptor, which could increase susceptibility to bacterial and viral pathogens and cause a more severe disease course. During the recent COVID-19 pandemic, mortality rates were higher in patients with risk factors such as advanced age, obesity, history of smoking, and underlying comorbidities, such as diabetes mellitus, arterial hypertension, and cancer, among others. It was also found that morbidity and mortality were higher in patients who had developed autoantibodies against type 1 interferon before their infection with COVID-19, as evidenced by studies of autoimmune polyendocrine type 1 syndrome.

A study of patients with COVID-19 found that 86% were hospitalized for COVID-19-related pneumonia, including 68% who were admitted to an intensive care unit, of whom 50% required mechanical ventilation and at least 18% died ^34^^,^^35^. Men, especially older adults, were found to have higher titers of antibodies against interferons in the population with a more severe course of the disease. In studies of these abnormal responses to interferon, Puel *et al*. found defects in 8 of the 13 loci associated with the induction and differentiation of type I interferons that depend on TLR3 and IRF7 ^35^.

Antibodies against interferons have also been found in other diseases, such as systemic lupus erythematosus. Several studies have shown that IgG-type antibodies to interferons can be found during the active disease or even up to two years after treatment. It is worth mentioning that the role of antibodies against interferons in systemic lupus erythematosus is somewhat contradictory since, when elevated, they play a fundamental role in the pathogenesis of the disease and are used as markers of its severity. However, IgG antibodies against type I interferons (IFN-a) are believed to act similarly to monoclonal antibodies used in systemic lupus erythematosus therapy. Nevertheless, high titers of antibodies to type II interferons (IFN-y) are thought to be associated with disease severity. It is critical to continue research in this field to understand better the role of IgG antibodies against interferons in systemic lupus erythematosus ^36^^,^^37^.

There have been several cases of people with symptoms like those of an HIV-immunocompromised patient but who tested negative for HIV. For this clinical scenario, Browne *et al*. coined the term “adult immunodeficiency syndrome” to refer to patients, typically older than 50 years, with high titers of neutralizing antibodies to IFN-y and associated immunodeficiency ^38^. People with this type of immunity to interferons are susceptible to various pathogens, including viruses and opportunistic bacteria, such as non- tuberculous ^2^^,^^6^. These cases have been reported in populations from Thailand, China, and the Philippines, who have presented primarily with mycobacterial abscesses ^33^^,^^35^.

Other microorganisms include Salmonella spp., *Cryptococcus neoformans, Histoplasmosis capsulatum, Burkholderia pseudomallei, Listeria* spp., and Talaromyces marneffe'r, the latter is also frequently present in disseminated diseases in patients with HIV, neoplasms, or prolonged corticosteroid use, raising importance in patients with adult immunodeficiency syndrome ^32^. This pathogen is transmitted by inhalation of conidia of the microorganism in the environment, later replicating within macrophages in the form of yeast and leading to localized infection in the lungs, skin, or even systemic dissemination, as has been reported in people with high titers of antibodies against IFN-y, who usually present more abrupt symptoms accompanied by pleural effusion and multiorgan involvement ^35^. Chen *et al*. reported that patients with positive antibodies against IFN-y developed complications, such as pleural effusion, despite receiving an optimal treatment ^33^.

## Autoimmunity against IL-6

In addition to the group of interferons, other cytokines are involved in autoimmunity, such as interleukin 6 (IL-6), known to play a fundamental role in the acute inflammatory response, hematopoiesis, and oncogenesis. This cytokine is produced by monocytes, macrophages, endothelial cells, fibroblasts, and hepatocytes in response to PAMP, IL-1, or TNF stimuli ^29^.

The receptor for IL-6 is either bound to the membrane or in soluble form. The activation mechanism occurs when IL-6 binds to its receptor and induces downstream signaling molecules-like Janus kinases (JAKs)-to recruit the receptor signal transducer and activator of transcription 3 (STAT3) or the mitogen-activated protein kinases (MAPK) via the receptor-associated molecule gp130. Subsequently, IL-6-inducible genes are transcribed ^2^ and produce, among others, proteins such as C-reactive protein (CRP), fibrinogen, and serum amyloid A, which are acute phase reactants. There is a pronounced increase in their synthesis during acute infectious processes, with enhanced intensity in bacterial infections. In addition, high concentrations of IL-6 stimulate adipose and white blood cells, inducing procalcitonin, an acute phase reactant serving as a specific marker for bacterial infections and a useful discriminatory marker in sepsis ^32^.

Regarding alterations in IL-6, patients with signaling defects in the MyD88/ IRAK/NF-k(3/Ik(3o pathway or the IL-6 receptor-dependent gp130/ZNF341/ STAT3 pathway are susceptible to staphylococcal infections ^39^. However, it has also been shown that patients with high levels of IL-6 antibodies are susceptible to pyogenic infections, like those with congenital impairments, due to the neutralization of IL-6 by IgG-type antibodies ^39^.

This has been demonstrated in four reported cases, in which all patients presented with severe staphylococcal infection, cellulitis, and abscess; one of these patients had a septic shock, but the common feature of all subjects was their low CRP levels despite presenting a clear clinical picture of severe bacterial infection. Other laboratory parameters were elevated, such as left-shift leukocytosis, lactic acid, and procalcitonin. These findings suggest that neutralization of IL-6 by IgG antibodies leads to more severe cases of pyogenic infections. This hypothesis is reinforced by the fact that the documented phenotype is very similar to that described in patients with homozygous IL6R mutations.

## Autoimmunity against the IL17 axis

The autoimmune polyendocrine syndrome type 1 is characterized by various endocrinopathies of autoimmune origin, such as adrenal insufficiency and hyperparathyroidism. This syndrome is caused by mutations in the *AIRE* gene, related to immunological tolerance in the thymus, and involved in the presentation of peripheral antigens in secondary lymphoid organs. However, some symptoms of this disease are due to high titers of autoantibodies against type I interferon, including IFN-a and IFN-ω, useful for its diagnosis ^37^.

Chronic mucocutaneous candidiasis is also frequent in patients with polyendocrine syndrome type 1 (APS-1). Previously, this infection establishment was associated with autoantibodies against type IIFN. However, recent studies found the implication of autoantibodies against other cytokines that support the immune response against *Candida albicans*, such as the IL-17 family (IL-17A, IL-17F, and IL-22) ^33^. This hypothesis was demonstrated in a study of 33 patients with autoimmune polyendocrine syndrome type 1 and chronic mucocutaneous candidiasis. Using flow cytometry and ELISA, the authors detected IgG autoantibodies against IL-17 in 22 patients, IL-17A in 31 patients, and IL-22 in 30 patients ^33^. Chronic mucocutaneous candidiasis is also favored in the setting of thymoma ^4^.

The mechanisms by which autoantibodies against IL-17 produce the fungal infection are not fully understood, but it is likely related to AIRE- dependent tolerance. IL-17 stimulation is required to produce antibodies against staphylococci ^33^, which increases the risk of severe staphylococcal infections in patients with anti-IL-17 antibodies, like those with deficits in the STAT3 signaling pathway.

## Autoimmunity against IL-1 and IL-2 receptors

Some cases of myocarditis associated with the messenger RNA vaccine against the SARS-CoV-2 virus have been described in children and adults between 14 and 79 years. These patients have neutralizing autoantibodies directed to the endogenous interleukin-1 receptor antagonist (IL-1 RA), responsible for inhibiting IL-1 signaling and inflammation. After receiving the second dose of the SARS-CoV-2 vaccine, patients with antibodies to IL-1 RA had an early onset of inflammatory symptoms but a milder course than those diagnosed with myocarditis, who did not have detectable autoantibodies. These cases may be associated with molecular mimicry between IL-1 RA and some viral antigens, including the spike protein ^6^.

Antibodies against IL-1 RA have also been observed in patients with lgG4- related disease, a fibroinflammatory disorder characterized by multiple organ involvement, tissue infiltration by plasma cells expressing lgG4, and storiform fibrosis ^40^. These autoantibodies are thought to promote proinflammatory and profibrotic factors, such as MMP-9 (Matrix Metallo Proteinase-9) and IL-33, in epithelial cells and fibroblasts stimulated by IL-1 a and the regulatory IL-1 RA, which plays a protective role. Dysregulation of these factors induce pathogenic inflammation and fibrosis, which could cause irreversible tissue damage and dysfunction. It is worth mentioning that high titers of antibodies against IL- 1 RA have been reported in patients with systemic lupus erythematosus and rheumatoid arthritis compared to healthy individuals ^41^. Autoantibodies against IL-1 RA are also found in a high proportion (50%) of patients suffering from multisystem inflammatory syndromes, frequent in COVID-19 cases, because of the elevated phosphorylation of the IL-1 RA isoform ^33^^,^^42^.

Regarding autoantibodies against IL-2, it is important to note that IL-2 modulates proliferation and clonal expansion of T lymphocytes. Therefore, this cytokine inhibition severely affects the activation of adaptive immunity ^9^. In addition, these autoantibodies could compromise the activity of regulatory T lymphocytes ^36^.

According to Shao *et al*., the role of autoantibodies against IL-2 is dual, depending on the antigenic region or epitope to which the autoantibodies are directed ^9^. They explained that, for example, when autoantibodies target an epitope in the IL-2 binding region with CD25, the immune response is enhanced. On the contrary, if they bind to another region, they could inhibit IL-2 binding to CD122, thereby suppressing the immune response ^9^.

## Future perspectives

Within the panorama we have reviewed, the growing interest in anticytokine autoantibodies in different medical conditions will continue its exponential growth in the following years. In the last year, several studies reported different findings regarding susceptibility to unusual infections in patients with anti-GM-CSF antibodies ^37^. Cheng *et al.* published an association of anti-IL23 autoantibodies with severe, persistent, recalcitrant, or opportunistic infections. In turn, Griffin *et al*. documented a phenocopy of an inborn error related to IL-10 signaling in a child with infantile-onset inflammatory bowel disease ^43^^,^^44^.

With these findings, two relevant considerations for the future emerge: first, it is critical to pursue active surveillance of the infection development in the context of increasing new biological treatment trends aiming to block specific cytokines; and second, it is necessary, in the clinical practice of specialties such as immunology, infectious diseases, and rheumatology, among others, to foster the capacity to detect anti-cytokine autoantibodies, since efforts such as those of Browne *et al*. to facilitate the performance of these assays are valuable and necessary for allowing a timely diagnosis of these cases ^38^.

## Conclusion

The autoimmune response against cytokines induces systematic alterations, which, in addition to exacerbating inflammatory responses, can increase susceptibility to infections for example, in autoimmunity against interferons, causing more severe conditions. The origin of this type of autoimmunity is unclear. However, it can result from immunodeficiencies. This hypothesis seems to be supported by the presence of autoantibodies before the development of the severe symptoms of some infections, such as those observed during the COVID-19 pandemic.
